# Favorable role of *IDH*1/2 mutations aided with *MGMT* promoter gene methylation in the outcome of patients with malignant glioma

**DOI:** 10.2144/fsoa-2020-0057

**Published:** 2020-12-09

**Authors:** Arshad A Pandith, Iqbal Qasim, Shahid M Baba, Aabid Koul, Wani Zahoor, Dil Afroze, Adil Lateef, Usma Manzoor, Ina A Bhat, Dheera Sanadhya, Abdul R Bhat, Altaf U Ramzan, Fozia Mohammad, Iqra Anwar

**Affiliations:** 1 Advanced Centre for Human Genetics, Sher-I-Kashmir Institute of Medical Sciences (SKIMS), Srinagar, J&K, 190011, India; 2 Immunology & Molecular Medicine, SKIMS, Srinagar, J&K, 190011, India; 3 Department of Pathology, SKIMS, Srinagar, J&K, 190011, India; 4 Department of Neurosurgery, SKIMS, Srinagar, J&K, 190011, India; 5 SRM University, Chennai, 603203, India; 6 Department of Biochemistry, School of life Sciences, Jaipur National University, 302004, India

**Keywords:** astrocytoma, GBM, *IDH*1/2, *MGMT* promoter gene, oligidendrioglioma, overall survival

## Abstract

**Aim:**

The implications of molecular biomarkers *IDH*1/2 mutations and *MGMT* gene promoter methylation were evaluated for prognostic outcome of glioma patients.

**Materials & methods:**

Glioma cases were analyzed for *IDH*1/2 mutations and *MGMT* promoter methylation by DNA sequencing and methylation-specific PCR, respectively.

**Results:**

Mutations found in *IDH*1/2 genes totaled 63.4% (N = 40) wherein *IDH*1 mutations were significantly associated with oligidendrioglioma (p = 0.005) and astrocytoma (p = 0.0002). *IDH*1 mutants presented more, 60.5% in *MGMT* promoter-methylated cases (p = 0.03). *IDH*1 mutant cases had better survival for glioblastoma and oligodendrioglioma (log-rank p = 0.01). Multivariate analysis confirmed better survival in *MGMT* methylation carriers (hazard ratio [HR]: 0.59; p = 0.031). Combination of both biomarkers showed better prognosis on temozolomide (p < 0.05).

**Conclusion:**

*IDH*1/2 mutations proved independent prognostic factors in glioma and associated with *MGMT* methylation for better survival.

## Lay abstract

Alterations in *IDH* gene and *MGMT* promoter methylation are associated with prediction for outcome of glioma (brain) tumors. We analyzed glioma cases, finding 63.4% had mutations in *IDH*1/2 genes. *IDH*1 mutations are associated with the glioma types oligidendrioglioma and astrocytoma. Patients with *IDH*1 mutation were seen more in defective *MGMT*-methylated cases. Glioblastoma and oligidendrioglioma patients with mutant *IDH*1 survive more than patients with the normal gene. A better prognosis was seen when both gene alterations were present in patients treated with a chemotherapy (temozolomide). Thus *IDH*1/2 mutations appear to be a good survival factor in glioma, in particular when associated with *MGMT* methylation.

## Graphical abstract

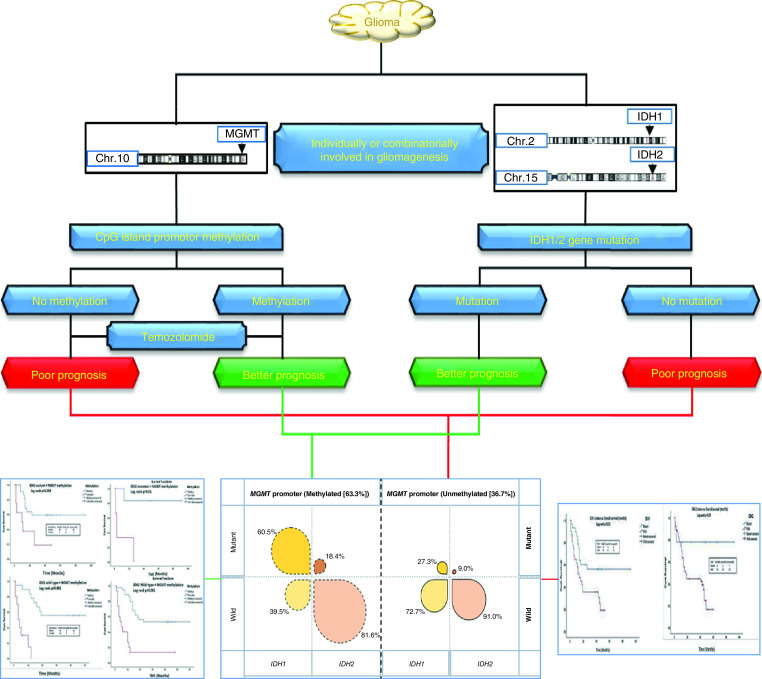

Glioma cases were analyzed for *IDH*1/2 mutations by DNA sequencing and *MGMT* methylation through methylation-specific PCR. Significant association was observed between *IDH*1 mutations and oligidendrioglioma (p = 0.005) and astrocytoma (p = 0.0002). Significantly, *IDH*1 mutants presented more, 60.5% in *MGMT*-methylated cases (p = 0.03). *IDH*1 mutant cases had better survival for glioblastoma and oligodendrioglioma (log-rank p = 0.01). A better prognosis was seen when both genetic biomarkers were compared with respect to temozolomide use (p < 0.05). Multivariate analysis showed *MGMT* promoter methylation emerged as an independent prognostic factor for overall survival. *IDH*1/2 mutations proved as independent prognostic factors in glioma and correlated with *MGMT* methylation for better survival.

Gliomas, the most predominant primary brain tumor, occur at an incidence of 1.6% and mortality rate of 2.5% globally with male:female ratio of age-standardized risk as 3.9:3.1 as per Global Cancer Statistics (2018). They demonstrate extensive diversity with respect to morphological site, genetic pattern and response to chemo-radiotherapy. Glioblastoma (GBM), the most prevalent malignant glioma, has very poor prognosis and develops aggressively with no indication of either primary GBM, or its progression from secondary GBM [1,2].

In addition to chromosomal changes, mostly chromosome 1p/19q deletion, prominent genetic and epigenetic alterations like *IDH* mutations and *MGMT* gene promoter methylation, respectively, have been found in malignant gliomas [3]. Brain tumorigenesis has witnessed a tremendous therapeutic advance, but the prognostic implications for glioma patients still remain unclear [4] with different survival ranges in different types of glioma [5].

Apart from various genetic events, the discovery of *IDH*1 and *IDH*2 mutations has recently been the most exciting recent discovery in understanding oncogenetic events of glioma. *IDH*1/2 are NADP^+^-dependent homodimeric isozymes that show substantial sequence analogy with very similar protein structure [6]. *IDH*1 is cytoplasmic component that is expressed prominently in the liver and other tissues, while *IDH*2 is entirely restricted to the mitochondria and shows vast expression in heart and muscle tissues, and lymphocytes [7]. Any mutation in *IDH*1 or -2 can result in enhanced oxidative stress by its mutagenic action that harms the DNA [8]. This phenomenon is sustained by an enhanced amount of DNA damage in *IDH*1-mutated malignant glioma cells and thereby *IDH*1/2 mutations operate as driver mutations in glioma carcinogenesis, although their primary role is still unexplored [9].

Both *IDH*1/2 mutations are more or less solely connected with glial-type phenotype of brain tumors and are detected in around 5% of primary and approximately 50% of secondary GBM that has been substantiated to confer an improved prognosis [10–12]. Furthermore, The Cancer Genome Atlas (TCGA) has permitted classification of diverse molecular variants of GBM with different outcomes with proneural showing a good prognosis, while neural, classical and mesenchymal exhibit a bad prognosis [13]. Recently, it has been suggested that the proneural variant is also linked to a better response to the antiangiogenic agent bevacizumab [14].

A fraction of malignant gliomas resist the chemotherapeutic agent temozolomide (TMZ), a potent reactive molecule that causes cell death [15,16]. TMZ is an alkylating agent that cross-links DNA via the DNA-repair enzyme O^6^-methylguanine-DNA methyltransferase (MGMT) [17]. *MGMT* repairs alkylating lesions of the DNA usually caused by the TMZ [18]. The cells with an intact *MGMT* gene exhibit drug resistance while therapeutic response to TMZ is improved in cancer cells with hypermethylated *MGMT*, which results in its repression resulting in the loss of *MGMT* protein expression [19]. This process is believed to render *MGMT* methylation a favorable prognostic advantage in glioma patients treated with alkylating agents [20]. Methylation of the *MGMT* promoter is seen as lower in frequency at around 35–45% of the cases of malignant gliomas (WHO grades III and IV), while it appears in approximately 80% of low-grade gliomas (WHO grade II) [20,21]. This group of glioma patients may have enhanced sensitivity to TMZ due to deficiency of the enzyme [22], which affects its clinical outcome.

Substantial evidence gleaned through various retrospective studies on glioma from numerous clinical trials has demonstrated that *IDH* mutations and *MGMT* gene promoter methylation have prognostic implications [23–25]. The tumoral *IDH1* mutations are now known to confer favorable prognosis with longer progression-free survival (PFS) and its mutated form in glioma patients (WHO grade III) sees a median prognosis of approximately 3.5 versus approximately 1.5 years for wild-type gliomas [24,26,27], while patients with *MGMT* promoter methylation benefit from chemotherapy and show superior prognosis [28–31]. A combination of *MGMT* promoter methylation and *IDH* mutation is now believed to significantly increase the overall survival (OS), and progression-free possibilities in glioma patients [29]. Recently, machine learning (ML)-based on set of algorithms has been used for diverse scenarios to allow precision in classification, progression, treatment and OS of certain diseases including glioma [32–35]. ML has been used on gliomas to predict OS using various datasets, especially TCGA [32], studying *IDH* mutation [35,36] and *MGMT* promoter methylation [37]. However, these studies still do not show improvement over the traditional statistical methods for clinical biomarkers [33].

The current study was designed to analyze the prognostic implications of *IDH*1/2 gene alterations and *MGMT* gene methylation for their individual as well as cumulative impact on outcome of malignant glioma patients with diverse histologies.

## Materials & methods

Sixty-three glioma patients that were diagnosed, histologically confirmed and previously untreated were included in this study. They attended the Departments of Medical Oncology and Neurosurgery of Sher-I-Kashmir Institute of Medical Sciences (SKIMS; J&K, India). Patients with malignant glioma were included after a written informed consent. Surgically resected tumor tissue samples were taken through stereotactic/open biopsy of brain tumors and biopsy samples were reviewed by two expert neuropatholigists to confirm the diagnosis of malignant glioma and ensure uniformity of classification criteria. Depending upon stage and cancer development, 5–10 mg of the tissue was collected in phosphate-buffered saline vials and stored at -70°C until further processing for DNA extraction, while a separate aliquot of each sample was stored and properly preserved. About 5 ml of peripheral blood was obtained from each patient in EDTA-containing vials (200 μl of 0.5 M, pH 8.0) for evaluating any germline mutation events as a mean for control for sporadic mutational events, and was stored at -20°C until use. Surgically resected tissue samples were collected directly into sterile vials containing chilled phosphate-buffered saline (pH 7.2) and were divided into two parts: one sent for histopathological diagnosis and the other one stored at -70°C for molecular investigations. Confirmed glioma tissues were used both for mutational analysis of *IDH*1/2 gene and *MGMT* gene promoter methylation. After pathological confirmation, glioma patients were given TMZ with radiotherapy as per the standard protocol. Patients were put on oral TMZ 100 mg/m^2^ per day for the duration of radiotherapy. After 3–4 weeks of radiotherapy, patients were put on TMZ 150–200 mg/m^2^ on days 1 through 5 in 28-day cycles for a maximum of 12 cycles.

### Patient selection

The study was conducted between 2013 and 2017 in the Department of Advanced Centre for Human Genetics, SKIMS. Patients who received previous cytotoxic chemotherapy or radiation were excluded from the analysis. There were no restrictions on age, sex, histology or stage, but patients with a prior history of cancer other than glioma were excluded from the study. Clinical information, including gender, age, tumor stage, tumor grade and histopathology were obtained from the review of patients/medical records. The patient cohort contained 47 male cases (74.6%) and 16 female (25.4%). Patients were grouped in two categories on the basis of age, <50 years and ≥50 years of age (Supplementary Figure 4). More cases were seen in age group <50 years (n = 33; 53.3%) than in ≥50 years (n = 30; 47.6%) with nearly equal ratio (median 56 years). Based on histopathological observation, there were 53.9% (n = 32) GBM cases, 22.2% (n = 14) oligodendrioglioma (OG), 22.2% (n = 14) astrocytoma and 4.7% (n = 03) belonged to other types of malignant glioma. Also more cases were found in clinical stages III and IV (n = 27; 42.9%) and (n = 29; 46.0%) than stages I and II (n = 2; 3.1%) and (n = 4; 6.3%) (Supplementary Table 1).

### DNA extraction

High-molecular-weight DNA was isolated by using *proteinase* K and phenol/chloroform extraction. The purity and concentration of DNA was estimated by measuring the absorbance at 260 and 280 nm and checked on 1% Nusieve agarose gel.

### Polymerase chain reaction

*IDH*1 *and IDH*2 genes (exon 4) were amplified using previously designed specific primers as *IDH*1-E × 4F-5′-GTTTAGGGTGTGCCAGTGC-3′, *IDH*1-E × 4R 5′-GTTGAGATGGACGC CTATTTG-3′ and *IDH*2-E × 4F-5′-GCTTGGGGTTCAAATTCTGG-3′, *IDH*2-E × 4R5′-GAA AGGAAAGCCACGAGACAG that resulted in an amplicon size of 658 and 534 bp, respectively. PCR amplification was performed in a 50-μl volume containing 250 ng of genomic DNA, 1x PCR buffer containing 25 mM MgCl_2_, 50 μM each of dATP, dGTP, dTTP and dCTP, 1.0 U of *Taq DNA polymerase* (Biotool Madrid, Spain) and 1 μM of forward and reverse primers (Sigma-Aldrich, IN, USA). After an initial denaturation at 95°C for 7 min, 35 cycles at 94°C for 30 s, annealing temperature (exon 2, 60°C;* exon* 4, 55°C) for 30 s, 72°C for 30 s and finally an extension temperature was performed for 7 min. The PCR products were run on 2% agarose gel and analyzed under an UV illuminator. The purified PCR amplicons of the tumor samples were used for direct DNA sequencing.

### DNA sequencing analysis

Mutation screening of each gene was performed by direct sequencing of genomic DNA in forward and reverse orientations using the Applied Biosystems Big Dye terminator reaction kit and the AB 3500 sequencing machine (Applied Biosystems, CA, USA).

### Bisulfite DNA modification & methylation-specific PCR

Tumor tissue-extracted DNA was modified by chemical bisulfite treatment (EZ DNA Methylation Kit, Zymo Research Corporation, CA, USA) and subjected to PCR using primers specific for methylated and modified unmethylated DNA [14] using previously performed protocol [38]. The final PCR products were run on a 2–3% agarose gel and ethidium bromide-stained gels yielded 81 and 93 bp methylated and unmethylated product, respectively. The gel bands were identified by using gel documentation system, Flourchem HD2 (Cell Bioscience, CA, USA). All the results were validated in two separate independent experiments where the researchers were kept unaware of the previous findings.

### Statistical analysis

Statistical analysis was performed using IBM Statistics SPSS software (version 23). The cases were compared using the Chi-square test for categoric variables (such as sex and age) of the demographic variables. Different tests for homogeneity of proportions including Chi square, Fisher’s exact test and Kaplan–Meier (KM) analysis to evaluate survival outcome probabilities were used to determine significance of the distribution patterns with respect to different clinico-analytical parameters. Frequency and percentage were calculated to express qualitative data. Odds ratio (OR) as an estimate of associative relative risk and the 95% CI was calculated using SPSS version 12 data analysis software. Univariate and multivariate Cox proportional hazard model was employed to determine the risk factors of any events. Statistical significance was set at the level of p < 0.05.

## Results

The overall frequency of mutations detected in both *IDH*1 and *IDH*2 genes was found to be 63.4% (40 of 63). When stratified, the frequency of *IDH*1 and *IDH*2 gene mutations was 49.20% (31 of 63) and 14.28% (9 of 63), respectively. Among 63 samples evaluated for mutations, one sample was found to harbor both *IDH*1 and *IDH*2 mutations in codon 132 and 172, respectively. All the mutations were heterozygous in nature and the majority (80.64%: 25/31) of *IDH1* mutations were G395A (Arg132His CGT >CAT), followed by three mutations as C394A (Arg132Ser, 9.6%), 02 as C394G (Arg132Gly, 6.45%) and 01 C394T (Arg132Cys, 3.22%) as shown in Table 1. Representative pictures of the electropherogram for the *IDH*1 gene exon 4 (codon 132) with different mutations identified in this study are given in Supplementary Figure 1.

**Table 1.** Frequency and nature of *IDH*1 and *IDH*2 mutations in glioma patients.

	Nucleotide change	Amino acid change	n (%)
*IDH*1 *Exon 4*	G395A (CAT) C394A (AGT) C394G (GGT) C394T (TGT)	Arg132His Arg132Ser Arg132Gly Arg132Cys	25/31 (80.64%) 03/31 (2.4%) 2/31 (1.9%)) 1/31 (1.1%) 31/63 (49.20%)
*IDH*2 *Exon 4*	G515A (AAG) G516T (AGT) G515T (ATG)	Arg172Lys Arg172Ser Arg172Met	06/09 (66.6%) 02/09 (22.2%) 01/09 (2.46%) 09/63 (14.28%)

*IDH*1 status was analyzed in each of the histological groups comprising astrocytoma, oligidendrioglioma and GBM tumors as summarized in Table 2. *IDH*1 mutations were most frequently present in tumors with OG (85.7%) and astrocytoma (71.4%), as compared with GBM (27.8%). A highly significant association was observed between *IDH*1 status with respect to OG (p = 0.0002) and astrocytoma (p = 0.005). Significantly, male patients showed higher frequency of *IDH*1 mutation than females (74.1 vs 25.8%; p = 0.0002) but was observed as nonsignificant among age group as well as vital status (p > 0.05). A noncanonical mutation at codon 90 of the *IDH*1 gene was found in only two cases but the functional analysis to evaluate its mechanistic role was not pursued as the frequency was very low. An identical series of samples was subsequently analyzed for *IDH*2 mutations. The frequencies of the *IDH*2 gene mutations are summarized in Table 2. The overall mutation detected in *IDH*2 domain exons 4 (codon 172) among 63 glioma patients were found to be 15% (9/63). *IDH*2 gene mutations were detected in 28% (2/14) astrocytoma cases, 28.5% (4/14) OG and GBM 9.3% (3/32). The description of the nature of mutations is given in Table 2, where all mutations were substitutions and heterozygous in nature. DNA sequence evaluation of the *IDH*2 exon in glioma samples revealed that all the nine somatic mutations were at residue R172: The R172 residue in *IDH*2 is the exact analog of the R132 residue in *IDH*1. Only nine tumors contained *IDH*2 mutation, including 06 as G515A (Arg172Lys, 66.66%), 02 as G516T (Arg172Ser, 22.22%) and one G515T (Arg172Met, 2.46%). Representative pictures of the electropherogram for the *IDH*2 gene exon 4 (codon 172) mutations identified in this study are given in Supplementary Figure 2. No significant association was found between the *IDH*2 mutations with respect to different clinicopathological parameters details of which are given in Table 2.

**Table 2.** Association of *IDH*1 and *IDH*2 gene mutations with clinical variables in glioma patients.

Variables	Glioma cases, n = 63 (%)	Wild	*IDH*1 mutation	p-value	Wild	*IDH*2 mutation	p-value
Age: ≥50 <50	30 (47.6) 33 (52.3)	18 (60.0) 14 (46.6)	12 (40.0) 19 (63.3)	0.2	27 (90.0) 27 (81.8)	03 (10.0) 06 (18.1)	0.4
Sex: Male Female	47 (74.6) 16 (25.3)	24 (51.0) 39 (82.9)	23 (74.1) 08 (25.8)	0.0002	40 (85.1) 14 (87.5)	07 (14.8) 02 (12.5)	0.99
Dwelling: Rural Urban	25 (39.6) 38 (60.3)	11 (44.0) 21 (55.2)	14 (56.0) 17 (44.7)	0.4	21 (84.0) 33 (86.8)	04 (16.0) 05 (13.2)	0.99
Tumor type: Glioblastoma Astrocytoma Oligidendrioglioma	32 (50.7) 14 (22.2) 14 (22.2)	25 (78.1) 04 (28.5) 02 (35.7)	07 (21.8) 10 (71.4) 12 (85.7)	0.2 0.005 0.0002	29 (90.6) 12 (85.7) 10 (71.4)	03 (9.4) 02 (14.3) 04 (28.6)	0.01 0.9 0.4
Grade: I/II III/IV	06 (9.5) 56 (88.8)	03 (50.0) 28 (50.0)	03 (50.0) 28 (50.0)	1.0	05 (83.3) 47 (83.9)	01 (16.7) 09 (16.1)	1.0
Vital status: Dead Alive	36 (57.1) 27 (42.8)	21 (58.3) 11 (40.7)	15 (41.6) 16 (59.2)	0.2	33 (91.6) 21 (77.7)	03 (9.4) 06 (22.3)	0.1

*MGMT* gene promoter methylation was observed in 60.3% (n = 38) of the glioma samples versus 39.7% (n = 22) unmethylated cases. Comprehensive data regarding *MGMT* gene promoter methylation status and expression have previously been presented by our group [38]. GBM cases showed 53.1% (n = 17) methylated sequences compared with 46.9% (n = 15) unmethylated, followed by astrocytoma at 64.2% (n = 9) and OG 85.7%. The overall pattern of methylation versus unmethylated status was observed to be significant in glioma cases (p < 0.05). Patients with a hypermethylated *MGMT* promoter depicted a significantly better OS and PFS of 40.0 and nearly 24 months, respectively, compared with 6.7 and 3.2 months when unmethylated (log-rank p > 0.05; Table 3). Compared with unmethylated cases, the effect of chemotherapy (TMZ therapy) on cases with *MGMT* methylated promoters resulted in a longer survival. As shown in Table 3, *IDH*1 mutants were observed in 23 (60.5%) of the 38 *MGMT* promoter-methylated glioma cases compared with wild-type 15 (39.5%), while six (27.3%) *IDH*1 mutants were found in 22 unmethylated cases versus 16 (72.7%) wild-type cases. The difference in *IDH*1 mutants among two groups was significant with OR: 3.6 (95% CI: 1.1–11.1), p = 0.03. Similarly, seven (18.4%) cases of mutant *IDH*2 presented in 38 methylated cases while two (9.0%) were seen in 22 unmethylated cases. Thirty-one (81.6%) cases of *IDH*2 wild-type were detected in methylated versus 20 (91.0%) in unmethylated cases with no association (p > 0.05). Furthermore, multivariate analysis of survival adjusted for age, gender, grade of disease and *IDH*1*/IDH*2 mutation confirmed better survival rates in *MGMT* promoter methylation carriers (HR: 0.59; 95% CI: 0.09–0.98; p = 0.031) (Table 4).

**Table 3.** Correlation of *IDH*1/2 mutational profile with *MGMT* promoter gene methylation status.

*IDH* mutation status	*MGMT* promoter methylation		OR (95% CI)	p-value
	Methylated n = 38	Unmethylated n = 22		
*IDH*1 mutant *IDH*1 wild	23 (60.5) 15 (39.5)	6 (27.3) 16 (72.7)	3.6 (1.1–11.1)	0.03
*IDH*2 mutant *IDH*2 wild	7 (18.4) 31 (81.6)	2 (9.0) 20 (91.0)	2.3 (0.42–11.9)	0.3
Parameter	Mean OS (months)	p-value	Mean PFS (months)	p-value
*MGMT* methylated *MGMT* unmethylated	40.09 6.75	0.000	23.9 3.2	0.000

OR: Odds ratio; OS: Overall survival; PFS: Progression-free survival.

**Table 4.** Multivariate analysis of glioma patients according to different clinical parameters and *MGMT* promoter methylation.

Variables	Hazard ratio	CI (95.0%)	p-value
Age: <50 ≥50	1.0 (Ref) 1.12	0.42–3.79	0.41
Sex: Male Female	1.0 (Ref) 1.57	0.71–4.91	0.73
Grade: I/II III/IV	1.0 (Ref) 0.95	0.35–2.55	0.51
*IDH1* wild: *IDH1* wild *IDH1* mutant	1.0 (Ref) 1.33	0.42–3.95	0.39
*IDH*2 wild: *IDH*1 wild *IDH*1 mutant	1.0 (Ref) 1.08	0.69–2.77	0.88
*MGMT* promoter methylation Unmethylated Methylated	1.0 (Ref) 0.59	0.09–0.98	0.031

The different histological subgroups of glioma, when stratified to analyze the relation between *IDH* mutational pattern and *MGMT* promoter methylation, did not show any significant difference as shown in Supplementary Table 2 (p < 0.05).

**Figure 1.**
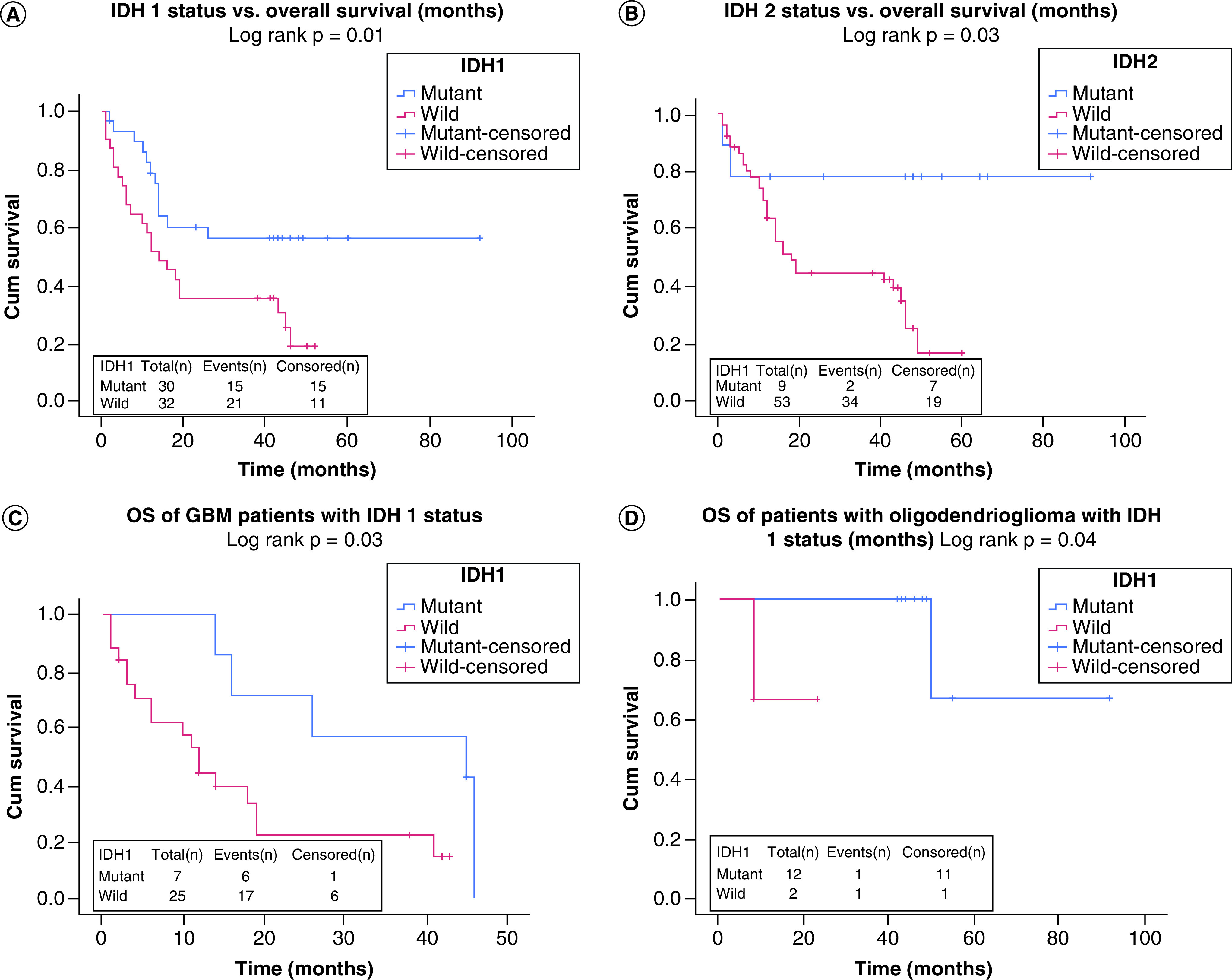
Overall survival of glioma patients when compared between *IDH* 1/2 mutants versus wild type by multivariate analyses. **(A)** OS of glioma patients with *IDH*1 mutational status. **(B)** OS of glioma patients with *IDH*2 mutational status. **(C)** Survival status of GBM patients with *IDH*1 status. **(D)** Survival status of oligidendrioglioma patients with *IDH*1 status. GBM: Glioblastoma; OS: Overall survival.

**Figure 2.**
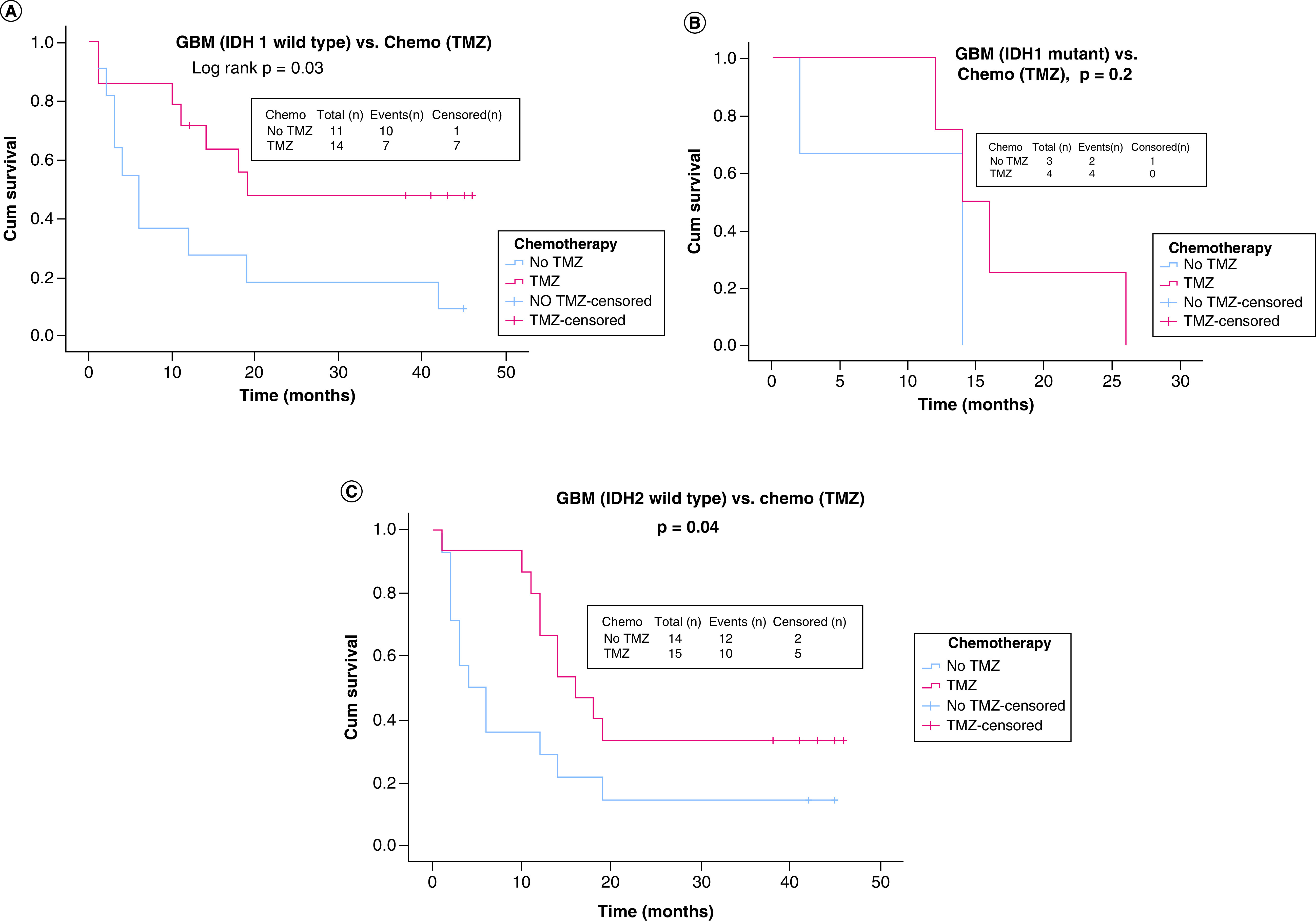
Overall survival of subgroups of glioma patients when compared between *IDH*1/2 status and chemotherapy (temozolomide). **(A)** OS of GBM patients with *IDH*1 wild-type status with respect to TMZ therapy. **(B)** OS of GBM patients with *IDH* 1 mutational status with respect to TMZ therapy. **(C)** OS of GBM patients with *IDH*2 wild-type status with respect to TMZ therapy. GBM: Glioblastoma; OS: Overall survival; TMZ: Temozolomide.

Using KM analysis, OS of glioma patients was evaluated when compared between *IDH*1/2 mutants versus wild type by multivariate analyses (Figure 1A–D). *IDH*1 mutants showed significantly higher OS of 56.8 (95% CI: 41.8–71.8) versus 22.7 months for *IDH*1 wild type (95% CI: 15.7–29.8) with (log rank p = 0.01) as shown in Figure 1A. In contrast, *IDH*2 mutants and wild type did not show any variation with respect to OS in overall glioma cases (log-rank p > 0.05), as depicted in Figure 1B. When GBM cases were stratified, *IDH*1 mutants revealed better survival of 34.0 versus 16.5 months for wild type (log-rank p = 0.03; Figure 1C) and an almost similar pattern was found for OG where the *IDH*1 mutant group showed better OS than wild type (log-rank p = 0.04; Figure 1D). Astrocytoma did not show any difference in OS in the glioma patients with different *IDH*1 status (log-rank p = 0.10). Interestingly, the *IDH*2 mutant/wild-type group did not show any difference in OS of GBM and OG patients (log-rank p > 0.05) but astrocytoma cases showed better survival in the *IDH*1 mutant group 51.5 (95% CI: 39.7–63.3) versus wild-type 21.8 months (14.1–29.5) with log rank p = 0.02. The impact of better survival by chemotherapy using TMZ irrespective of *IDH*1 mutation status was observed in the glioma cases. Both the *IDH*1 wild type (12.2 months TMZ not used vs 32.7 months TMZ used) and mutant *IDH*1 (18.8 months TMZ not used vs 57.1 months TMZ used) showed significant difference with respect to use of TMZ chemotherapy and in fact showed better OS when chemotherapy was used across all histological types of glioma patients (log-rank p < 0.05). On the other hand, when glioma was histologically sub-grouped to analyze the impact of chemotherapy using TMZ, GBM showed significantly better survival in both wild-type groups of *IDH*1 (13.0 vs 27.5 months; log-rank p = 0.03; Figure 2A) and *IDH* 2 (23.8 vs 11.7 months; log-rank p = 0.04; Figure 2B) as compared with mutants (10.0–17.0 months for *IDH*1; Figure 2C). Astrocytoma showed less difference of OS among patients when TMZ was used with *IDH*1 mutant phenotype (26.5 for no TMZ group vs 31.5 months for TMZ group: log-rank p = 0.9), while on the other hand, patients with the same histology had relatively better OS for *IDH* wild-type phenotypes (6.0 for no TMZ group vs 28.5 months for TMZ group: log-rank p = 0.09). Furthermore, when different combinations of *IDH*1/2 status were associated with *MGMT* promoter methylation, significantly better prognosis was observed in terms of survival of patients irrespective of both positive and negative mutation status of *IDH* gene, as shown in Supplementary Figure 3A–D.

## Discussion

A robust use of genetic biomarkers has been reported in the recent past for the treatment and management of malignant gliomas from diagnostics to the classification of tumors. These biomarkers such as *IDH* gene mutation and *MGMT* promoter methylation status help to predict prognosis and could predict response to specific chemotherapies, thus holding promise for the safe and specific treatment of malignant glioma.

Different types of *IDH* mutations alter normal catalytic enzyme *IDH* activity and are related to a specific glioma CpG island methylator phenotype, which is characterized by extensive hypermethylated loci. This phenotype occurs in low-grade gliomas, a younger age group of patients and has been substantiated to confer a favorable prognostic outcome [27].

Mutated *IDH*1 diminishes the action of NADPH that is necessary for cellular defense against oxidative stress, leading to tumorigenesis owing to oxidative DNA damage [39]. The frequency of mutations detected in *IDH*1/2, at 63.4% (N = 40), is in agreement with Parsons *et al.* [40]. Furthermore, our results confirm other studies conducted across the globe that have found that this mutation is present in approximately 80% of grade 2–3 gliomas and secondary GBM [10,41,42]. *IDH*1 mutations known to date are single amino acid missense mutations like arginine 132 (R132) and a related residue in the *IDH*2 gene (R172) [10,13,41]. Our study has found all the mutations in these respective codons, but a novel mutation was also detected in codon 90 of the *IDH*1 gene in two cases (3.1%). This non-canonical mutation has not been reported so far from any of the investigation across the globe either in glioma or any other cancer. Since its frequency was very low, it could not be correlated statistically with any other parameters like age, gender and OS or PFS. Although we did not study the functional analysis of this mutation, it seems that there are certain unexplored molecular alterations, which may be related to certain ethnic groups or any other occupational or related carcinogenic exposure.

Interestingly, we found both *IDH*1/2 genes mostly prevalent in two subtypes of gliomas: astrocytoma and oligodendrogliomas with a frequency of 71.4 and 85.7% for *IDH*1 and 14.28 and 28.5% for *IDH*2, respectively. A similar scenario of mutational spectrum has been reported from other studies conducted in different regions of the world where grade II/III astrocytomas and OG harbor 80% of mutations and approximately 15% are found in GBMs [40]. The identification of these mutations is prevalent for the majority of WHO grades II/III astrocytic, oligodendroglial and oligoastrocytic gliomas [43]. It is plausible from the reports including our study that *IDH* mutations in malignant gliomas lead to different clinical course and are genetically distinct from gliomas that harbor wild-type *IDH* genes. Our report seems to be in agreement with a previous report where *IDH* mutations are substantiated as an early event in the development of a glioma [42]. In this study, the detection of the majority of *IDH*1/2 mutations in oligoastrocytoma tumors supports this conjecture.

Epigenetic variation in DNA methylation in *MGMT* gene and its pivotal role in chemotherapy provides a stage for the prediction of better survival as confirmed in our previous study [38]. In the current study, we found a significant correlation between *MGMT* promoter methylation and *IDH*1 status in glioma cases (p = 0.03). We found *IDH*1 mutants presented 3.6-fold more, 60.5% in *MGMT* methylated glioma cases as compared with 27.3% in unmethylated cases and in this context, Martin *et al.* [44] found a similar scenario of correlation with the same frequency of *IDH*1 mutations (62%) in *MGMT* promoter-methylated tumors compared with 10% in unmethylated ones. Yet another study also highlighted the same association with similar results where *IDH*1 mutations 58 versus 60.5% (in our study) were found in methylated compared with 26 versus 27.3% in the unmethylated group [11]. The above finding does not conform to a study conducted in Germany by Wick *et al.* [45] where two biomarkers showed independent prognostic significance. Through multivariate analysis, we found *MGMT* promoter methylation emerged as an independent prognostic factor for OS. On the contrary, *IDH*2 mutational pattern and *MGMT* methylation status showed comparable frequency in both methylated and unmethylated *MGMT* gene for same series of glioma patients and therefore, did not correlate significantly (p > 0.05).

KM analysis revealed significantly better OS (56.8 vs 22.7 months for *IDH*1 wild type; log-rank p = 0.01) for glioma patients with *IDH*1 mutations (Figure 1A). In contrast, differential *IDH*2 status revealed no variation with respect to OS of glioma cases (Figure 1B). The study conducted by Parsons *et al.* [40] demonstrated a similar observation where glioma patients presented with better outcome that carried the *IDH*1 mutation. Furthermore, Yan *et al.* [10] established the same finding in another larger group of glioma patients while incorporating *IDH*2 mutation status. Our study is in partial agreement with Yan *et al.* [10], who confirmed that GBM with an *IDH*1 mutation had a median OS of 31 versus 34 months (our study), which was significantly better than the 15 versus 16 months (our study) in patients with wild-type *IDH*1 (log-rank p < 0.05). *IDH*2 mutations did not show any impact on survival in GBM cases as depicted by Yan *et al.* [10], but interestingly showed better survival in astrocytoma cases (51.5 vs wild-type 21.8 months, log-rank p = 0.02). In 2010, Martin *et al.* validated the same findings where glioma cases carrying *IDH*1 mutation showed a prognostic implication for longer survival. In addition, the same study [44] is in agreement with ours regarding the better outcome of OG patients carrying *IDH*1 mutants. The impact on survival by TMZ therapy irrespective of *IDH*1 mutation status was observed to be better in glioma cases. Both the *IDH*1 wild type and mutant showed significant difference with respect to use of TMZ chemotherapy and in fact displayed better OS across all histological types of glioma patients (log-rank p < 0.05: Supplementary Figure 2A). A multicenter study conducted in 2010 [44] reported no suggestion that *IDH*1 mutations can predict the outcome for use of TMZ therapy. In yet another report on TMZ therapy, no association was substantiated for the outcome of glioma patients and *IDH* mutational status [46]. Yet, it still remains to be seen whether both mutational spectrums of *IDH*1/2 predict response of malignant gliomas to chemotherapy. A recent RTOG 9402 trial has found that patients with 1p/19q non-co-deleted *IDH*1-mutant anaplastic gliomas have better survival after chemotherapy [47]. In EORTC 26951 reports, *IDH*1-mutants showed more benefit from procarbazine, CCNU (lomustine) and vincristine (PCV) chemotherapy, but were not statistically significant. The *IDH*1 mutation spectrum was retrospectively analyzed in a subset of patients in the NOA-04 study [45], but was not found to have a predictive response to chemotherapy.

Furthermore, the impact of chemotherapy in GBM using TMZ showed significantly better survival in both wild-type groups of *IDH*1/2 than mutants (Figure 2B). A subgroup like astrocytoma shared less difference of OS among patients when TMZ was used with *IDH*1 mutant (log rank = 0.9) while the same histology patients had relatively better OS for *IDH* wild-type phenotypes (log-rank p = 0.09). This finding is in stark contrast with the study conducted by Sanson *et al.* [48] for *IDH*1 (codon 132 only) wherein OS of grade IV gliomas (primary GBM) was better in *IDH*1-mutated GBMs than nonmutated ones (OS, 27.4 vs 14 months, respectively). The results of all these investigations, taken together, validate that invariably better survival in glioma patients with respect to *IDH*1 mutant tumors can mainly be attributed to less aggressive oncogenic mode and is less likely due to outcome of treatment by chemotherapy. On the other hand, *MGMT* gene promoter-methylated tumors show significant improved OS. Furthermore, it was seen in our study that different combinations of *IDH*1/2 mutational statuses (irrespective of either wild or mutated) and *MGMT* methylation showed a better prognosis for survival in glioma patients. Similarly, Houillier *et al.* [49] and Kazuhiro *et al.* [50] have reported the same findings where *MGMT* promoter methylation and *IDH* mutation status were associated with a better response and survival with TMZ therapy. Mutations in the *IDH* gene accumulate 2-hydroxyglutarate causing hypermethylation of DNA sequences, alongside a few other consequences [51], that strongly associate to originate a biochemical event in tumorigenesis to initiate methylation of *MGMT* gene sequences at promoter. This probably delineates a molecular root for the association between *IDH*1 mutations and *MGMT* methylation [11].

## Conclusion

*IDH*1/2 mutations represent less aggressive oncogenic mode and independently prove to be a better prognostic factor for survival in glioma patients. *IDH*1 mutational spectrum correlated with *MGMT* promoter gene methylation and points toward very strong prognostic implications for better survival. Furthermore, a better prognosis for survival in glioma cases was indicated for *IDH* gene status irrespective of positive or negative with *MGMT* promoter methylation. Evaluation of different molecular biomarkers is a very good option and has the potential to predict the outcome for glioma patients treated with chemotherapy.

## Future perspective

Research at the molecular and translational level in glioma has considerably progressed our understanding of the genetic pathogenesis of glioma. Numerous molecular and cellular biomarkers are now recognized that aptly act in diagnosis, prognosis and in the prediction of glioma behavior and outcome, thus their clinical application has helped to improve its treatment and management. The prominent biomarkers in glioma management currently include *MGMT* promoter methylation, EGFR amplification, 1p/19q co-deletion, *IDH* mutation and B-Raf. *IDH* mutation and *MGMT* promoter methylation either individually or in combination are robustly prognostic in glioma. Besides the implication of *IDH* mutations as a prognostic factor in grade II gliomas, they designate the foundation of glioblastoma from a previous lower-grade lesion. On the other hand, *MGMT* promoter methylation shows promise both as prognostic and predictive markers to benefit patients on chemotherapy. The future for glioma treatment shows promise with the use of antibodies against the *IDH1* R132H mutation in different neuro-oncology centers to differentiate diffuse infiltrating gliomas. Furthermore, development of more IDH antibodies for the other specific mutations implicated in glioma are currently in progress.

*MGMT* gene promoter methylation has its prognostic power, but there is an urgent need to unravel the molecular mechanisms underlying resistance to chemotherapy, as this is yet to be fully understood. A fraction of patients show resistance to TMZ despite favorable *MGMT* gene expression and the mechanism behind this is now believed to involve certain other genetic aspects like miRNAs that have a plausible impact on regulation of *MGMT* gene expression. Future lines of investigation include miRNAs that are being investigated whose deregulation can alter *MGMT* gene expression and subsequently leads to resistance. Investigating the acquired chemotherapeutic resistance conferred by these molecules carries the potential to improve the survival rate of patients. Earlier, an operational signature of survival association has been built-up to envisage drug targets for specific clones within a tumor and thus efficient study tools like ML augment diagnosis and predict efficacy of radio-chemotherapy. Besides on the methodological side, more sensitive *MGMT* protein detection by modern tools will likely help to improve protein validation for successful TMZ therapy and its response in glioma.

**Summary points**

- The current study analyzed prognostic implications of *IDH*1/2 gene alterations and *MGMT* promoter gene methylation for their individual as well as cumulative significance in outcome of malignant glioma.
- Glioma cases were analyzed for *IDH*1/2 mutations by DNA sequencing and *MGMT* methylation through methylation-specific PCR.
- Mutations found in *IDH*1/2 genes totaled 63.4% in glioma cases (N = 40).
- A significant association was observed between *IDH*1 mutations and oligidendrioglioma (p = 0.005) and astrocytoma (p = 0.0002).
- *MGMT* gene promoter methylation was observed in 60.3% (n = 38) of glioma samples wherein overall methylated versus unmethylated cases showed significant difference in glioma patients (p < 0.05).
- Significantly, *IDH*1 mutants presented more, at 60.5%, in *MGMT*-methylated cases (p = 0.03). *IDH*1 mutant cases had better survival for glioblastoma and oligodendrioglioma (log-rank p = 0.01).
- Patients with hypermethylated *MGMT* promoter depicted a better overall survival and progression-free survival (40.0 and 24 vs 6.7 and 3.2 months in unmethylated ones; log-rank p > 0.05).
- Glioblastoma and oligodendrioglioma cases with *IDH*1 mutations revealed better survival against wild type (log-rank p < 0.05).
- Better survival by chemotherapy (temozolomide) was observed irrespective of *IDH*1/2 mutation status.
- In conclusion, *IDH*1/2 mutations represent less aggressive oncogenic mode and prove to be independently a better prognostic factor for survival in glioma patients.
- The *IDH*1 mutational spectrum in association with *MGMT* gene methylation points toward very strong prognostic implications for better survival.
- Evaluation of different molecular biomarkers like *IDH* gene alterations and *MGMT* promoter methylation has the potential to predict the outcome of glioma patients treated with chemotherapy.
