# Supplementary material for: Favorable role of IDH1/2 mutations aided with MGMT promoter gene methylation in the outcome of patients with malignant glioma
Source: Future Sci OA. 2020 Dec 9;7(3):FSO663. doi: 10.2144/fsoa-2020-0057 (PMC7849969; doi:10.2144/fsoa-2020-0057)
Supplement: Supplementary file 1 [file fsoa-07-663-s1.docx]

| ***R132S Wild (394 CGT)*** |  | ***R132S Mutant (394 CGT>AGT)*** |
| --- | --- | --- |
| ***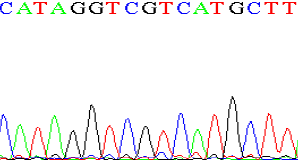*** |  | ***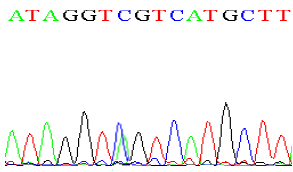*** |

Supplementary Figure 1: DNA Sequencing Electropherograms depicting *IDH1* mutation

| ***R172K Wild* (*515AGG*)** |  | ***R172K Mutant (515 AGG>AAG)*** |
| --- | --- | --- |
| ***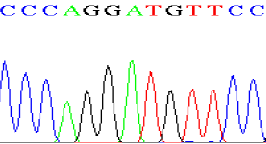*** |  | ***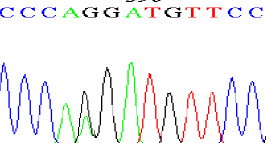*** |

Supplementary Figure 2: DNA Sequencing Electropherograms depicting *IDH2* mutation

**Supplementary Figure 4: Age distribution of glioma patients**
