# Supplementary figures and images for: Favorable role of IDH1/2 mutations aided with MGMT promoter gene methylation in the outcome of patients with malignant glioma
Source: Future Sci OA. 2020 Dec 9;7(3):FSO663. doi: 10.2144/fsoa-2020-0057 (PMC7849969; doi:10.2144/fsoa-2020-0057)

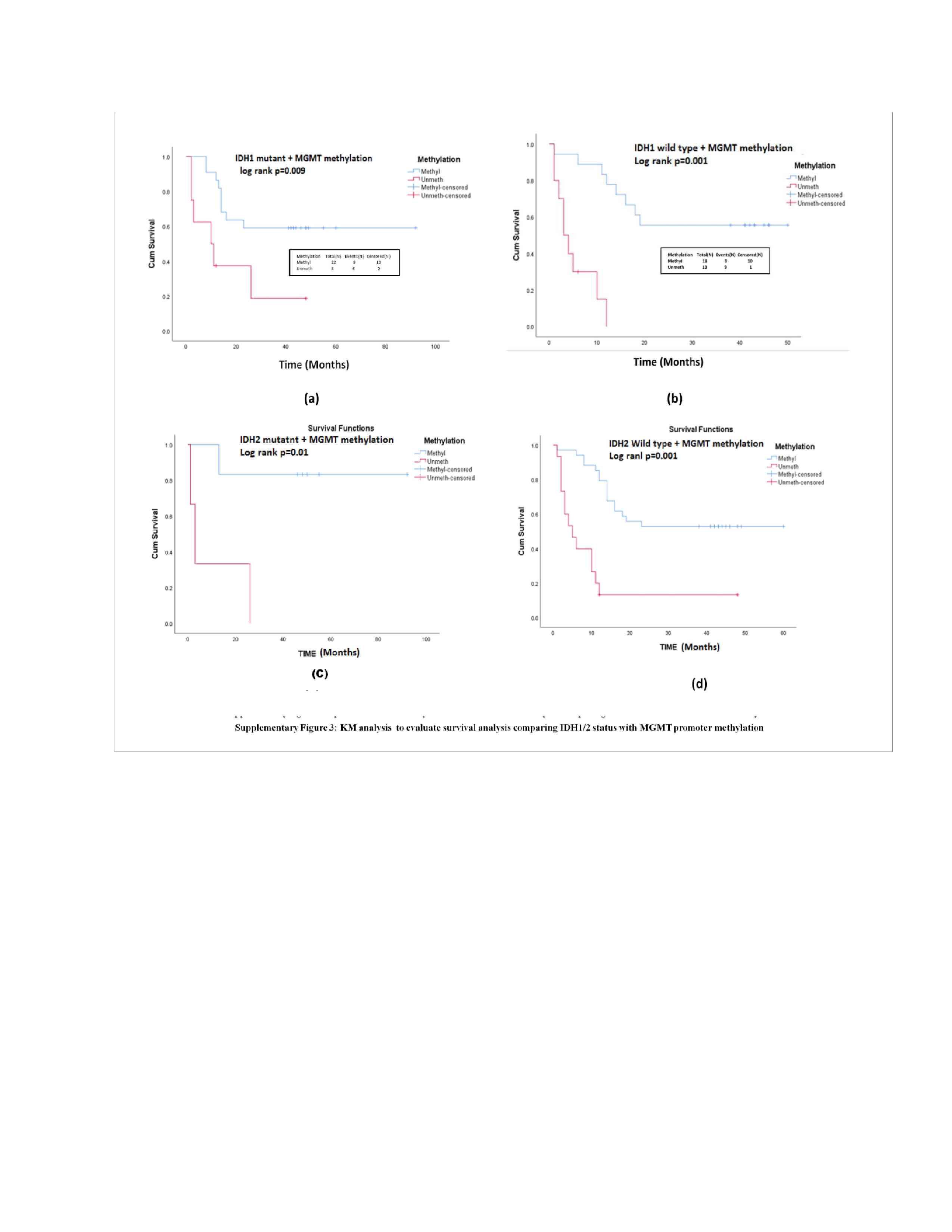

Supplement: Supplementary file 2 [file fsoa-07-663-s2.jpg]
