# Supplementary material for: Favorable role of IDH1/2 mutations aided with MGMT promoter gene methylation in the outcome of patients with malignant glioma
Source: Future Sci OA. 2020 Dec 9;7(3):FSO663. doi: 10.2144/fsoa-2020-0057 (PMC7849969; doi:10.2144/fsoa-2020-0057)
Supplement: Supplementary file 3 [file fsoa-07-663-s3.docx]

**Supplementary Table 1: Distribution analysis of selected demographics factors in glioma cases**

| **Demographic Feature** | **Glioma cases n=63** | **(%)** |
| --- | --- | --- |
| **Age**  ≥50  <50 | 30  33 | (47.6)  (53.3) |
| **Sex**  Male  Female | 47  16 | (74.6)  (25.4) |
| **Residence**  Rural  Urban | 25  38 | (39.7)  (60.3) |
| **Tumor Type**  Glioblastoma  Astrocytoma  Oligidendrioglioma  Others | 32  14  14  03 | (53.9)  (22.2)  (22.2)  (4.7) |
| **Grade**  I  II  III  IV | 2  4  27  29 | (3.1)  (6.3)  (42.9)  (46.0) |
| **Vital Status**  Dead  Alive | 36  27 | (57.1)  (42.9) |

**Supplementary Table 2: Relation between *IDH*1/2 mutations with different subgroups of malignant glioma**

| **Variables** | ***IDH*1/2**  **Wild type** | ***IDH*1/2**  **Mutant** | **P value** |
| --- | --- | --- | --- |
| **Glioblastoma**  Methylated  Unmethylated | 33  22 | 4  6 | 0.2 |
| **Astrocytoma**  Methylated  Unmethylated | 10  6 | 7  5 | 0.9 |
| **Oligidendrioglioma**  Methylated  Unmethylated | 9  2 | 15  1 | 0.5 |
| **Low grade**  Methylated  Unmethylated | 9  0 | 3  0 | 1.0 |
| **High grade**  Methylated  Unmethylated | 44  29 | 24  10 | 0.2 |
